# Knowledge, attitude, and practice regarding cancer pain management in Southwest China: a two-center cross-sectional study

**DOI:** 10.3389/fpubh.2026.1871970

**Published:** 2026-07-22

**Authors:** Xuexing Wang, Youxian Dou, Junyu Liu, Chao Song, Xingxing Tang, Guozhong Zhou, Zuchun Luo

**Affiliations:** 1 Department of Oncology, Anning First People's Hospital Affiliated to Kunming University of Science and Technology, Anning, China; 2 Faculty of Medicine, Kunming University of Science and Technology, Kunming, Yunnan, China; 3 Pain Medicine Department, Anning First People's Hospital Affiliated to Kunming University of Science and Technology, Anning, China; 4 Department of Thoracic Surgery, The Third People’s Hospital of Honghe Prefecture & Honghe Prefecture Cancer Hospital, Gejiu, Honghe Hani and Yi Autonomous Prefecture, Yunnan, China; 5 Student Affairs Office, The First Affiliated Hospital of Guangxi Medical University, Nanning, China

**Keywords:** analgesic use, cancer pain, knowledge, attitude, practice, pain management, rural health, Southwest China

## Abstract

**Background:**

Cancer pain management depends partly on patients’ knowledge, attitudes, and practices (KAP), but evidence from Southwest China remains limited. This study assessed cancer pain-related KAP, identified factors associated with Practice, and explored whether Attitude statistically accounted for the association between Knowledge and Practice.

**Methods:**

A two-center cross-sectional study was conducted among 523 patients with cancer-related pain at two tertiary hospitals in Southwest China. Data were collected using a self-developed KAP questionnaire, and domain scores were standardized to a 0–100 scale. Preliminary psychometric properties were assessed using internal consistency, item-level analyses, and confirmatory factor analysis. Factors associated with Practice were examined using multivariable linear regression. Exploratory indirect-effect analyses were performed using the PROCESS macro.

**Results:**

The mean standardized scores were 61.3 ± 32.6 for Knowledge, 62.0 ± 21.1 for Attitude, and 53.3 ± 28.9 for Practice; 51.6% of participants had poor Practice scores. Higher Knowledge score and peri-urban or urban residence were associated with higher Practice scores, whereas greater current pain intensity, age ≥60 years, and female sex were associated with lower Practice scores. Stage IV disease was positively associated with Practice after adjustment and remained generally consistent in the education-adjusted sensitivity model, although unadjusted analyses showed the opposite direction. Neither unadjusted nor covariate-adjusted exploratory indirect-effect analysis supported a statistically significant indirect association through Attitude.

**Conclusion:**

Among tertiary-hospital patients with cancer pain in Southwest China, Practice was comparatively lower than Knowledge and Attitude. Improving cancer pain-management practice may require attention to knowledge-to-action translation, symptom burden, and care-context factors rather than knowledge or attitudes alone. Future prospective, multicenter, and community-based studies are needed to validate the questionnaire and clarify temporal relationships among KAP domains.

## 1 Introduction

Cancer remains a major global and national health burden. GLOBOCAN 2022 estimated nearly 20 million new cancer cases and 9.7 million cancer deaths worldwide in 2022, and projects more than 35 million annual new cases by 2050 (1). In China, an estimated 4.82 million new cancer cases and 2.57 million cancer deaths occurred in 2022 (2). Pain is among the most common and distressing symptoms experienced by patients with cancer; a recent systematic review reported an overall cancer pain prevalence of 44.5%, with 30.6% of patients experiencing moderate-to-severe pain (3). Although evidence-based guidelines and opioid recommendations are available for cancer pain management (4, 5), analgesic undertreatment remains common, with updated systematic evidence suggesting that approximately 40% of patients with cancer pain receive analgesic treatment inadequate for pain intensity (6).

The gap between available pain-management strategies and actual pain control is shaped by barriers at the health-system, clinician, patient, and caregiver levels. From the patient perspective, insufficient knowledge, negative attitudes toward analgesics, fear of addiction or adverse effects, reluctance to report pain, and irregular analgesic use may interfere with effective cancer pain management (7, 8). In China, studies among physicians and other healthcare providers have reported gaps in cancer pain assessment, opioid-related knowledge, and pain-management practice (9, 10). More recent patient-focused evidence has further highlighted opioid-related concerns and patient-related barriers in cancer pain management (11). Evidence from Chinese cancer populations has also shown that pain-related knowledge, attitudes, caregiver influence, and analgesic adherence are associated with pain-management outcomes (12–14). These findings suggest that improving cancer pain management requires attention not only to prescriptions and clinical protocols, but also to patients’ understanding, beliefs, and day-to-day self-management behaviors.

The Knowledge, Attitude, and Practice (KAP) framework provides a pragmatic approach for examining modifiable cognitive, attitudinal, and behavioral factors related to health management (15). In cancer pain care, a KAP-based assessment can help identify whether patients understand key principles of pain reporting and analgesic use, how they perceive cancer pain and opioid treatment, and how they manage pain in daily practice. However, associations among Knowledge, Attitude, and Practice in cross-sectional studies should be interpreted as statistical relationships rather than evidence of temporal ordering or causal pathways.

Southwest China, particularly Yunnan Province, has distinctive geographic, ethnic, socioeconomic, and healthcare-access characteristics that may influence cancer pain management. Previous studies have reported urban–rural disparities in healthcare utilization among Chinese patients with cancer, health inequities among rural residents in Southwest China, and differences in cancer-related quality of life or health awareness across ethnic and rural populations in Yunnan and Southwest China (16–19). Nevertheless, cancer pain-related KAP among patients in this region remains insufficiently characterized. Therefore, this two-center cross-sectional study aimed to assess cancer pain-related KAP among patients receiving care at two tertiary hospitals in Southwest China, identify factors associated with pain-management practice, and explore the indirect association between knowledge and practice through attitude. Given the cross-sectional design, the indirect-effect analysis was exploratory and should not be interpreted as evidence of causal mediation (20).

## 2 Materials and methods

### 2.1 Study design and participants

This two-center cross-sectional study was conducted in the oncology, pain-management, and palliative-care departments of two tertiary hospitals in Southwest China between January 1 and December 1, 2025. Participants were recruited by convenience sampling. Eligible patients were adults aged 18 years or older with a confirmed cancer diagnosis and cancer-related pain, who were able to understand the questionnaire and provide written informed consent. Cancer-related pain was defined as pain attributed to the tumor, cancer treatment, or cancer-related complications based on clinical assessment by the attending physician. Patients were excluded if they had severe cognitive impairment, major communication difficulties, acute medical instability, or pain primarily unrelated to cancer.

Potentially eligible patients were first identified by attending physicians or nurses in the participating departments according to the predefined eligibility criteria. Trained research assistants then approached eligible patients, explained the study purpose and confidentiality procedures, and obtained written informed consent. Data were collected mainly through face-to-face interviews using a structured KAP questionnaire to improve completeness and accommodate patients with different literacy levels. Patients who were able and preferred to complete the questionnaire independently were allowed to do so. Each survey took approximately 15–20 min. Participation was voluntary, and refusal or withdrawal did not affect medical care. Questionnaires with more than 20% missing data on the KAP items were considered invalid and excluded from the final analysis.

**Figure 1:**
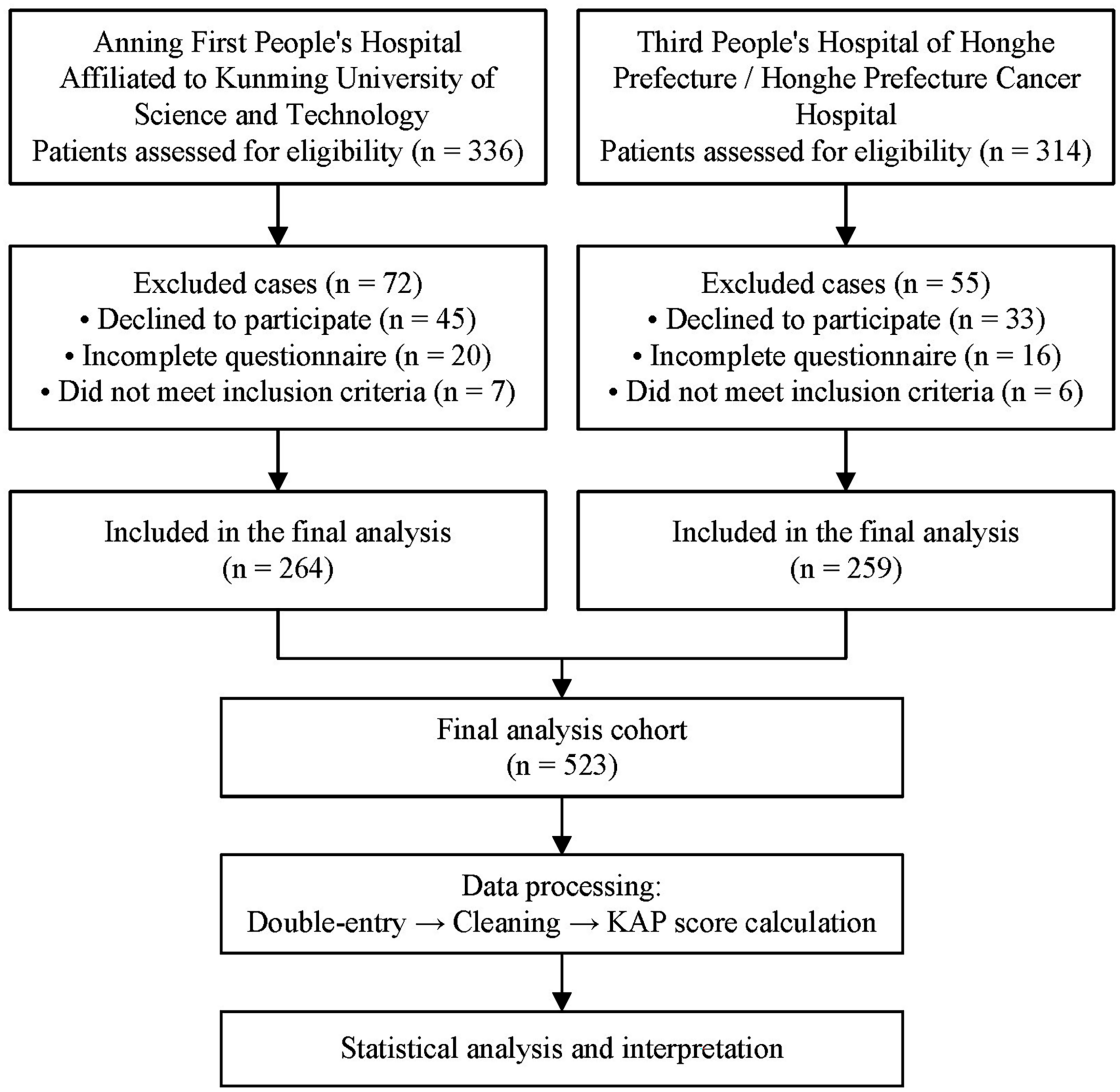
Participant recruitment and analytic workflow. KAP, Knowledge, Attitude, and Practice.

The study was conducted in accordance with the Declaration of Helsinki and was approved by the Institutional Review Board of Anning First People’s Hospital Affiliated to Kunming University of Science and Technology (IRB Approval No. 2025–043-01), with the Third People’s Hospital of Honghe Prefecture/Honghe Prefecture Cancer Hospital participating as a collaborating center under this approval. Written informed consent was obtained from all participants. The overall recruitment process, data-collection workflow, quality-control procedures, and analytic process are shown in Figure 1.

### 2.2 Sample size estimation

The sample size was estimated *a priori* using G*Power version 3.1 for multiple linear regression (21). Assuming a medium effect size (f^2^ = 0.15), a two-sided *α* level of 0.05, 80% statistical power, and up to 10 predictor variables, the minimum required sample size was 118 participants. To account for invalid questionnaires and improve the stability of model estimation, a larger sample was targeted. The final analytic sample included 523 participants, exceeding the minimum requirement for the primary multivariable regression analysis. Because the sample-size calculation was based on the regression model, the confirmatory factor analysis, subgroup comparisons, and exploratory indirect-effect analysis should be interpreted as supportive or hypothesis-generating analyses rather than independently powered confirmatory analyses.

### 2.3 Survey instrument

The study-specific KAP questionnaire was developed by the research team based on the KAP framework and previous literature on cancer pain management, patient-related barriers, analgesic adherence, and pain-management outcomes (7, 8, 11–15). The initial item pool was refined through expert consultation and pilot testing. An expert panel comprising specialists in oncology, pain management, palliative care nursing, and health psychology reviewed the draft questionnaire for relevance, clarity, cultural appropriateness, and content coverage. The average scale-level content validity index (S-CVI/Ave) was 0.92 (22). A pilot test was then conducted among 35 patients with cancer pain who met the eligibility criteria. Cognitive debriefing was used to identify ambiguous, difficult, or culturally sensitive wording, and minor revisions were made accordingly. Data from the pilot participants were not included in the final analysis.

The final questionnaire comprised 34 items in four sections. The first section included 10 sociodemographic and clinical items: age, sex, ethnicity, residence, education level, cancer diagnosis, cancer stage, pain duration, current pain intensity at the time of survey, and regular analgesic use. Current pain intensity was assessed using the 0–10 Numeric Rating Scale, with 0 indicating no pain and 10 indicating the worst imaginable pain. Cancer diagnosis and cancer stage were initially recorded using broad questionnaire categories and were subsequently checked against medical records or physician assessment for analytic classification. The patient-reported cancer-stage categories were used as initial self-report information and were not solely relied upon to distinguish stage III from stage IV disease. For statistical analyses, the analytically classified cancer-stage variable was categorized as stage I–III versus stage IV. When patient-reported staging information was unclear or insufficiently specific, medical-record or physician-verified staging information was used for classification. Regular analgesic use was defined as patient-reported regular or scheduled use of analgesics according to medical advice. The second section assessed cancer pain-related knowledge using 8 Yes/No/Unclear items. The third section assessed attitude toward cancer pain management using 8 items rated on a 5-point Likert scale, including four reverse-worded items: A2, A3, A4, and A6. The fourth section assessed pain-management practice using 8 items rated on a 5-point Likert scale, including one reverse-worded item, P8. All reverse-worded items were reverse-coded before item-level analyses, reliability estimation, subscale scoring, and subsequent statistical analyses, so that higher scores consistently indicated greater knowledge, more positive attitudes, or more appropriate pain-management practices.

Because the questionnaire was self-developed, its psychometric evaluation was considered preliminary. Internal consistency was assessed using the Kuder–Richardson 20 coefficient for the dichotomously scored Knowledge subscale and Cronbach’s *α* for the Likert-type Attitude and Practice subscales. To improve transparency and reproducibility, the full questionnaire is provided in Supplementary material 3, including the original Chinese version used for data collection and an English translation for publication reference. Additional item-level analyses were performed, including item-level distributions, descriptive statistics, corrected item-total correlations, reliability coefficients if item deleted, and floor and ceiling effects. The item-level psychometric results are reported in Supplementary material 1 (Supplementary Tables S1–S7). The absence of test–retest reliability, criterion validity, convergent validity, discriminant validity, measurement invariance, responsiveness, and validation in an independent sample was acknowledged as a limitation and should be addressed in future validation studies.

### 2.4 Data processing and scoring

All data were double-entered into EpiData 3.1 and cross-checked for accuracy; after reconciliation, the final discrepancy rate was less than 0.1%. Questionnaires with more than 20% missing data on the KAP items were excluded. The overall item-missing rate was 1.5%. Missing values were handled using multiple imputation with five imputed datasets, given the low overall missing rate (23). The imputation model included sociodemographic variables, clinical variables, and KAP subscale scores used in the analytic models, and pooled estimates were used for subsequent analyses.

Knowledge items were scored as 1 for a correct answer and 0 for an incorrect or “Unclear” answer, yielding a raw score range of 0–8. Attitude and Practice items were rated on 5-point Likert scales, with raw scores ranging from 8 to 40 for each subscale. All reverse-worded items were reverse-coded before scoring, so that higher scores consistently indicated greater knowledge, more positive attitudes, or more appropriate pain-management practices. To allow comparison across domains, raw scores were transformed to a 0–100 scale using the formula: standardized score = [(raw score - minimum possible score) / (maximum possible score - minimum possible score)] × 100. For descriptive purposes only, standardized scores were categorized as poor (<60), moderate (60 to <80), or good (≥80), consistent with Bloom-type percentage cutoffs used in recent oncology KAP studies (24, 25). These categories were not used in the multivariable regression or exploratory indirect-effect analyses.

Preliminary structural validity was evaluated using confirmatory factor analysis in IBM SPSS AMOS 26.0. A theoretically specified three-correlated-domain CFA model was examined, in which the 24 scored KAP items loaded onto their corresponding domains: Knowledge, Attitude, and Practice. This model was used only to examine whether the item responses were broadly consistent with the expected domain structure of the questionnaire; it was not used to justify the calculation or interpretation of an overall KAP score. The main descriptive, regression, and exploratory indirect-effect analyses therefore continued to use the three standardized domain scores separately. Model fit was assessed using χ^2^/df, CFI, TLI, RMSEA, and SRMR. Values of χ^2^/df < 3, CFI and TLI > 0.90, and RMSEA and SRMR <0.08 were considered indicative of acceptable fit, while recognizing that fit-index cutoffs should be interpreted as guidelines rather than rigid criteria (26). Because the questionnaire included both dichotomously scored and Likert-type items, and because AMOS-based CFA was used only as a preliminary structural check, the CFA results were interpreted cautiously as preliminary structural evidence rather than definitive validation evidence. Future validation should use methods better suited to categorical or ordinal data, such as analyses based on tetrachoric or polychoric correlations and robust weighted or diagonally weighted least-squares estimators (27). A detailed variable-coding table used for the statistical analyses is provided in Supplementary material 2.

### 2.5 Statistical analysis

All statistical analyses were performed using IBM SPSS Statistics for Windows, version 26.0 (IBM Corp., Armonk, NY, USA). Confirmatory factor analysis was conducted using IBM SPSS AMOS 26.0, and exploratory indirect-effect analysis was conducted using the PROCESS macro for SPSS, version 4.1 (20). All tests were two-sided, and *p* < 0.05 was considered statistically significant.

Categorical variables were summarized as frequencies (n) and percentages (%). Continuous variables were summarized as means and standard deviations or medians and interquartile ranges, as appropriate. Distributional patterns were assessed by visual inspection of histograms and Q–Q plots. Standardized Knowledge, Attitude, and Practice scores were described using both mean-based and median-based statistics. For each categorical patient characteristic, differences in standardized KAP scores were examined using the Mann–Whitney U test for two-group comparisons and the Kruskal–Wallis H test for multi-group comparisons. No adjustment for multiple comparisons was applied because these subgroup comparisons were descriptive and hypothesis-generating. Spearman’s rank correlation coefficients were calculated to examine associations among continuous standardized Knowledge, Attitude, and Practice scores.

Multivariable ordinary least-squares linear regression was used to identify factors associated with standardized Practice score. The primary model included Knowledge score, residence, current pain intensity at the time of survey, age group (<60 vs. ≥ 60 years), cancer stage (stage I–III vs. stage IV), and sex. These variables were selected *a priori* based on the KAP framework, clinical plausibility, prior evidence, and the need to maintain a parsimonious and interpretable primary model. All selected variables were entered simultaneously using forced entry, and no automated stepwise selection was used. Categorical variables were entered as dummy variables, with rural residence, mild current pain intensity, age <60 years, stage I–III disease, and male sex as reference categories.

Regular analgesic use was not included in the primary model because of its potential conceptual overlap with medication-related behaviors in the Practice domain, and was instead examined in a sensitivity model. Education level, ethnicity, and pain duration were examined in descriptive and univariate analyses. Education level was additionally included in a sensitivity model by adding it to the primary multivariable model, with low education used as the reference category. Recruitment center was not included in the regression models because no participant-level recruitment-center variable was available for analysis in the final de-identified analytic dataset. Therefore, potential residual confounding by recruitment center or center-level care patterns was considered in the interpretation of the findings and in the study limitations.

The regression model reported unstandardized coefficients, standard errors, 95% confidence intervals, standardized coefficients, *p* values, variance inflation factors, R^2^, and adjusted R^2^. Residual diagnostics were assessed using Q–Q plots and plots of standardized residuals against predicted values. Because the Practice score was bounded between 0 and 100 and residual diagnostics suggested some deviation from normality, a sensitivity analysis using heteroscedasticity-consistent robust standard errors was conducted (28). Regression diagnostics and sensitivity analyses are provided in Supplementary material 2 (Supplementary Tables S8–S13; Supplementary Figures S1–S3).

An exploratory indirect-effect analysis was conducted using PROCESS Model 4 to examine the indirect association through Attitude between Knowledge and Practice (20). Knowledge, Attitude, and Practice were entered as continuous standardized scores on a 0–100 scale. Both unadjusted and covariate-adjusted models were performed. The covariate-adjusted model included residence, current pain intensity, age group, sex, and cancer stage, consistent with the primary multivariable regression model. The indirect association was estimated using 5,000 bootstrap resamples, and statistical significance was assessed based on whether the 95% bootstrap confidence interval excluded zero. Given the cross-sectional design, this analysis was intended as a descriptive decomposition of observed associations and was not interpreted as evidence of temporal ordering or causal mediation.

## 3 Results

### 3.1 Participant flow and baseline characteristics

The participant recruitment and analytic workflow are shown in Figure 1. A total of 650 patients with cancer-related pain were assessed for eligibility, including 336 from Anning First People’s Hospital Affiliated to Kunming University of Science and Technology and 314 from the Third People’s Hospital of Honghe Prefecture/Honghe Prefecture Cancer Hospital. After excluding 127 patients, 523 were included in the final analysis, giving an inclusion proportion of 80.5%. The median age was 66 years (IQR, 59–72; range, 18–89), and 292 participants (55.8%) were female. Most participants had stage IV disease (418, 79.9%), pain duration longer than 6 months (261, 49.9%), and moderate or severe current pain intensity at the time of survey (458, 87.6%). Regular analgesic use was reported by 367 patients (70.2%). Gastrointestinal cancer, breast cancer, gynecological cancer, and lung cancer each accounted for approximately one-fifth of the sample, whereas liver cancer and other cancers accounted for smaller proportions. No missing or unclear cancer diagnosis was recorded. Baseline sociodemographic and clinical characteristics are summarized in Table 1.

**Table 1:** Baseline sociodemographic and clinical characteristics of patients with cancer pain (*n* = 523).

Characteristics	n (%)
Socio-demographic characteristics	
Age, years	
≤50	36 (6.9)
51–59	111 (21.2)
60–70	207 (39.6)
≥71	169 (32.3)
Sex	
Male	231 (44.2)
Female	292 (55.8)
Ethnicity	
Han	366 (70.0)
Ethnic minority	157 (30.0)
Residence	
Rural residence	209 (40.0)
Peri-urban residence	105 (20.1)
Urban residence	209 (40.0)
Education level*	
Low education	261 (49.9)
Intermediate education	232 (44.4)
High education	30 (5.7)
Clinical characteristics	
Cancer diagnosis	
Lung cancer	104 (19.9)
Liver cancer	52 (9.9)
Gastrointestinal cancer	105 (20.1)
Breast cancer	105 (20.1)
Gynecological cancer	105 (20.1)
Other cancers	52 (9.9)
Cancer stage	
Stage I–III	105 (20.1)
Stage IV	418 (79.9)
Duration of pain	
<1 month	52 (9.9)
1–3 months	53 (10.1)
3–6 months	157 (30.0)
>6 months	261 (49.9)
Current pain intensity, NRS**	
Mild pain, 1–3	65 (12.4)
Moderate pain, 4–6	238 (45.5)
Severe pain, 7–10	220 (42.1)
Regular analgesic use***	
Yes	367 (70.2)
No	156 (29.8)

Values are presented as n (%). *Education level was categorized as follows: low education, junior high school or below; intermediate education, senior high school or vocational school; high education, college/university or above. **NRS, Numeric Rating Scale. Current pain intensity was assessed at the time of survey using a 0–10 scale, with 0 indicating no pain and 10 indicating the worst imaginable pain. ***Regular analgesic use was defined as patient-reported regular or scheduled use of analgesics according to medical advice. Age was described using four categories in Table 1 and dichotomized as <60 vs ≥60 years for univariate and multivariable analyses. Cancer stage was initially collected using broad questionnaire categories and was subsequently checked against medical records or physician assessment where available. For the final analytic dataset, cancer stage was categorized as stage I–III and stage IV for analysis. No participant reported a current pain intensity score of 0 at the time of survey. Cancer diagnosis was recorded as a mutually exclusive baseline clinical variable. No missing or unclear cancer diagnosis was recorded.

### 3.2 Preliminary psychometric properties of the KAP questionnaire

Preliminary psychometric results of the self-developed KAP questionnaire are provided in Supplementary material 1, including item-level distributions, corrected item-total correlations, internal consistency indices, and floor/ceiling effects. The Knowledge subscale showed good internal consistency, with a KR-20 coefficient of 0.854. The Attitude and Practice subscales showed high internal consistency, with Cronbach’s *α* values of 0.956 and 0.977, respectively. Corrected item-total correlations were positive for all retained items, and several items showed potential floor or ceiling effects. These high internal consistency estimates should be interpreted alongside the item-level results, because they may also indicate some item redundancy. The theoretically specified three-correlated-domain CFA model showed acceptable preliminary fit: χ^2^/df = 2.58, CFI = 0.92, TLI = 0.91, RMSEA = 0.06 (90% CI, 0.05–0.07), and SRMR = 0.05. This finding was interpreted as preliminary support for the expected KAP domain structure, rather than as justification for using an overall KAP score. Overall, these findings provide preliminary psychometric evidence in the present sample, rather than full external validation.

### 3.3 Standardized KAP domain scores

Standardized KAP scores are summarized in Table 2. The mean standardized scores for Knowledge, Attitude, and Practice were 61.3 ± 32.6, 62.0 ± 21.1, and 53.3 ± 28.9, respectively. The corresponding median scores were 62.5 (Q1, Q3: 37.5, 87.5), 65.6 (Q1, Q3: 56.2, 75.0), and 59.4 (Q1, Q3: 28.1, 81.2), respectively. Overall, Practice scores were lower than Knowledge and Attitude scores, suggesting an apparent knowledge–practice gap.

**Table 2:** Standardized scores and descriptive categories of cancer pain KAP dimensions among patients (*n* = 523).

Dimension	Mean ± SD	Median (Q1, Q3)	Poor, n (%)	Moderate, n (%)	Good, n (%)
Knowledge	61.3 ± 32.6	62.5 (37.5, 87.5)	215 (41.1)	87 (16.6)	221 (42.3)
Attitude	62.0 ± 21.1	65.6 (56.2, 75.0)	173 (33.1)	252 (48.2)	98 (18.7)
Practice	53.3 ± 28.9	59.4 (28.1, 81.2)	270 (51.6)	119 (22.8)	134 (25.6)

KAP, Knowledge, Attitude, and Practice; SD, standard deviation; Q1, first quartile; Q3, third quartile. Values are presented as mean ± SD, median (Q1, Q3), or n (%). All domain scores were standardized to a 0–100 scale. Poor, moderate, and good were defined as standardized scores <60, 60 to <80, and ≥80, respectively, and were used for descriptive purposes only. These categories were not used in the multivariable regression or exploratory indirect-effect analyses.

For descriptive interpretation, standardized domain scores were categorized as poor, moderate, and good. In the Knowledge dimension, 215 patients (41.1%) were classified as poor, 87 (16.6%) as moderate, and 221 (42.3%) as good. In the Attitude dimension, 173 patients (33.1%) were classified as poor, 252 (48.2%) as moderate, and 98 (18.7%) as good. In contrast, 270 patients (51.6%) were classified as having poor Practice scores, whereas 119 (22.8%) and 134 (25.6%) were classified as moderate and good, respectively. These descriptive distributions indicate that Practice remained the weakest domain. Detailed score distributions and descriptive categories are shown in Table 2.

### 3.4 Univariate associations with KAP scores

Univariate analyses are shown in Table 3. Knowledge scores differed significantly by residence, education level, cancer stage, pain duration, current pain intensity, and regular analgesic use, but not by age, sex, or ethnicity. Attitude scores differed significantly by age, sex, residence, education level, cancer stage, pain duration, current pain intensity, and regular analgesic use, but not by ethnicity. Practice scores differed significantly across all examined sociodemographic and clinical characteristics. Because regular analgesic use conceptually overlapped with medication-related behaviors in the Practice domain, this variable was retained for descriptive and sensitivity analyses only and was not included in the primary multivariable regression model.

**Table 3:** Univariate comparisons of standardized cancer pain knowledge, attitude, and practice scores across patient characteristics (*n* = 523).

Characteristics	Knowledge score, median (Q1, Q3)	*p* value	Attitude score, median (Q1, Q3)	*p* value	Practice score, median (Q1, Q3)	*p* value
Socio-demographic characteristics						
Age, years		0.353		<0.001		<0.001
<60 (*n* = 147)	62.5 (50.0, 75.0)		68.8 (59.4, 75.0)		59.4 (46.9, 71.9)	
≥60 (*n* = 376)	62.5 (37.5, 75.0)		65.6 (53.1, 71.9)		53.1 (37.5, 65.6)	
Sex		0.303		0.013		0.034
Male (*n* = 231)	62.5 (50.0, 75.0)		65.6 (56.3, 75.0)		56.3 (40.6, 71.9)	
Female (*n* = 292)	62.5 (50.0, 75.0)		62.5 (53.1, 71.9)		53.1 (37.5, 65.6)	
Ethnicity		0.171		0.545		<0.001
Han (*n* = 366)	62.5 (50.0, 75.0)		65.6 (56.3, 75.0)		56.3 (40.6, 71.9)	
Ethnic minority (*n* = 157)	62.5 (50.0, 75.0)		62.5 (53.1, 71.9)		53.1 (37.5, 65.6)	
Residence		<0.001		<0.001		<0.001
Rural residence (*n* = 209)	37.5 (25.0, 50.0)		59.4 (50.0, 65.6)		40.6 (28.1, 53.1)	
Peri-urban residence (*n* = 105)	62.5 (50.0, 75.0)		65.6 (59.4, 71.9)		56.3 (43.8, 68.8)	
Urban residence (*n* = 209)	75.0 (62.5, 87.5)		71.9 (65.6, 78.1)		68.8 (56.3, 78.1)	
Education level		<0.001		<0.001		<0.001
Low education (*n* = 261)	50.0 (37.5, 62.5)		62.5 (53.1, 68.8)		46.9 (34.4, 59.4)	
Intermediate education (*n* = 232)	75.0 (62.5, 87.5)		68.8 (62.5, 75.0)		62.5 (50.0, 75.0)	
High education (*n* = 30)	75.0 (62.5, 87.5)		75.0 (68.8, 81.3)		71.9 (59.4, 81.3)	
Clinical characteristics						
Cancer stage		0.017		<0.001		0.022
Stage I–III (*n* = 105)	75.0 (50.0, 87.5)		68.8 (59.4, 75.0)		62.5 (46.9, 75.0)	
Stage IV (*n* = 418)	62.5 (37.5, 75.0)		65.6 (56.3, 71.9)		53.1 (37.5, 68.8)	
Duration of pain		<0.001		<0.001		<0.001
<1 month (*n* = 52)	50.0 (37.5, 62.5)		59.4 (50.0, 68.8)		53.1 (40.6, 65.6)	
1–3 months (*n* = 53)	62.5 (50.0, 75.0)		65.6 (56.3, 75.0)		56.3 (43.8, 68.8)	
3–6 months (*n* = 157)	75.0 (62.5, 87.5)		68.8 (62.5, 78.1)		62.5 (50.0, 75.0)	
>6 months (*n* = 261)	62.5 (37.5, 75.0)		65.6 (56.3, 71.9)		50.0 (34.4, 65.6)	
Current pain intensity, NRS		<0.001		<0.001		<0.001
Mild pain, 1–3 (*n* = 65)	62.5 (50.0, 75.0)		65.6 (56.3, 75.0)		62.5 (50.0, 75.0)	
Moderate pain, 4–6 (*n* = 238)	75.0 (62.5, 87.5)		68.8 (62.5, 78.1)		65.6 (53.1, 78.1)	
Severe pain, 7–10 (*n* = 220)	50.0 (25.0, 62.5)		62.5 (53.1, 68.8)		40.6 (28.1, 53.1)	
Regular analgesic use		<0.001		<0.001		<0.001
No (*n* = 156)	25.0 (0.0, 25.0)		56.2 (25.0, 62.5)		12.5 (3.1, 40.6)	
Yes (*n* = 367)	87.5 (62.5, 87.5)		65.6 (62.5, 81.2)		65.6 (43.8, 81.2)	

Values are presented as median (Q1, Q3). Mann–Whitney U tests were used for two-group comparisons, and Kruskal–Wallis H tests were used for multi-group comparisons. *p* values are shown on the row of each characteristic. No adjustment for multiple comparisons was applied because these analyses were descriptive and hypothesis-generating. KAP, Knowledge, Attitude, and Practice; NRS, Numeric Rating Scale. Current pain intensity was assessed at the time of survey using the 0–10 Numeric Rating Scale. Cancer stage was initially collected using broad questionnaire categories and was subsequently checked against medical records or physician assessment where available. For the final analytic dataset, cancer stage was categorized as stage I–III and stage IV for analysis.

### 3.5 Correlations among KAP dimensions

All three standardized KAP domain scores were positively correlated with each other (all *p* < 0.001; Table 4). The strongest correlation was observed between Knowledge and Practice (*ρ* = 0.773), followed by Knowledge and Attitude (ρ = 0.562). The correlation between Attitude and Practice was weaker but remained positive (ρ = 0.416).

**Table 4:** Spearman correlations among knowledge, attitude, and practice scores (*n* = 523).

Dimension	Knowledge score	Attitude score	Practice score
Knowledge score	1	0.562*	0.773*
Attitude score	0.562*	1	0.416*
Practice score	0.773*	0.416*	1

Values are Spearman’s rank correlation coefficients (ρ). *p < 0.001, two-tailed.

### 3.6 Multivariable analysis of factors associated with practice

The primary multivariable linear regression model is presented in Table 5. The model explained 78.9% of the variance in standardized Practice scores (R^2^ = 0.789, adjusted R^2^ = 0.785; *F* = 239.69, *p* < 0.001). Higher Knowledge score was associated with higher Practice score (B = 0.29, 95% CI, 0.23 to 0.35; *p* < 0.001). Compared with rural residence, peri-urban residence (B = 20.14, 95% CI, 15.67 to 24.61; *p* < 0.001) and urban residence (B = 28.56, 95% CI, 24.77 to 32.36; *p* < 0.001) were associated with higher Practice scores. Compared with mild current pain intensity, moderate pain (B = −7.95, 95% CI, −13.62 to −2.28; *p* = 0.006) and severe pain (B = −19.15, 95% CI, −25.89 to −12.41; *p* < 0.001) were associated with lower Practice scores in the ordinary least-squares model. Age ≥60 years (B = −9.16, 95% CI, −13.23 to −5.08; *p* < 0.001) and female sex (B = −4.64, 95% CI, −7.28 to −2.00; *p* < 0.001) were also associated with lower Practice scores. Stage IV disease was positively associated with Practice score after adjustment (B = 13.47, 95% CI, 7.64 to 19.29; *p* < 0.001).

**Table 5:** Primary multivariable linear regression model for factors associated with standardized practice score (*n* = 523).

Variable	B	SE	95% CI	Standardized β	*p* value	VIF
Intercept	30.07	2.39	25.39 to 34.76	—	< 0.001	—
Knowledge score	0.29	0.03	0.23 to 0.35	0.328	< 0.001	3.20
Residence						
Peri-urban vs. rural	20.14	2.28	15.67 to 24.61	0.280	< 0.001	2.43
Urban vs. rural	28.56	1.93	24.77 to 32.36	0.485	< 0.001	2.62
Current pain intensity						
Moderate vs. mild	−7.95	2.89	−13.62 to −2.28	−0.137	0.006	6.03
Severe vs. mild	−19.15	3.43	−25.89 to −12.41	−0.328	< 0.001	8.38
Age group						
≥ 60 vs. < 60 years	−9.16	2.07	−13.23 to −5.08	−0.143	< 0.001	2.54
Cancer stage						
Stage IV vs. stage I–III	13.47	2.96	7.64 to 19.29	0.187	< 0.001	4.12
Sex						
Female vs. male	−4.64	1.34	−7.28 to −2.00	−0.080	< 0.001	1.30

B, unstandardized coefficient; SE, standard error; CI, confidence interval; β, standardized coefficient; VIF, variance inflation factor; NRS, Numeric Rating Scale. The dependent variable was the Practice score standardized to a 0–100 scale. Categorical variables were entered as dummy variables, with the reference categories shown in the variable column. Current pain intensity was assessed at the time of survey using the 0–10 Numeric Rating Scale and was categorized as mild pain, NRS 1–3; moderate pain, NRS 4–6; and severe pain, NRS 7–10. The primary model included Knowledge score, residence, current pain intensity, age group, cancer stage, and sex. Regular analgesic use was not included in the primary model because of its potential conceptual overlap with medication-related behaviors included in the Practice dimension. Model R^2^ = 0.789; adjusted R^2^ = 0.785; F = 239.69, *p* < 0.001. All VIF values were below the conventional threshold of 10, suggesting no evidence of severe multicollinearity. However, the VIF values for the current pain-intensity variables were relatively high, indicating possible moderate collinearity; therefore, coefficients related to pain intensity and cancer stage should be interpreted cautiously.

VIF values ranged from 1.30 to 8.38. Although all values were below the conventional threshold of 10, the VIFs for current pain-intensity categories were relatively high, suggesting possible moderate collinearity. Therefore, the coefficients for pain intensity and cancer stage were interpreted cautiously.

Sensitivity analyses and regression diagnostics are provided in Supplementary material 2 (Supplementary Tables S8–S13; Supplementary Figures S1–S3). After adding regular analgesic use to the model, regular analgesic use was positively associated with Practice score (B = 14.60, 95% CI, 10.01 to 19.19; *p* < 0.001), while the directions of the core associations remained generally unchanged. In the robust standard-error sensitivity analysis, the results were also generally consistent, except that moderate current pain intensity was no longer statistically significant. Supplementary unadjusted analyses showed that patients with stage IV disease had lower Practice scores than those with stage I–III disease before covariate adjustment; this direction change after adjustment was therefore reported descriptively and interpreted cautiously. In the additional sensitivity model including education level, the main findings remained generally unchanged; stage IV disease remained positively associated with Practice score after additional adjustment for education level (B = 13.07, 95% CI, 7.16 to 18.98; *p* < 0.001).

### 3.7 Unadjusted and covariate-adjusted exploratory indirect-effect analyses through attitude

To examine whether there was an indirect association between Knowledge and Practice through Attitude, unadjusted and covariate-adjusted exploratory indirect-effect analyses were conducted using the Hayes PROCESS macro (Model 4), with Knowledge, Attitude, and Practice entered as continuous standardized scores on a 0–100 scale. The covariate-adjusted model included residence, current pain intensity, age group, sex, and cancer stage. The results are summarized in Table 6.

**Table 6:** Unadjusted and covariate-adjusted exploratory indirect-effect analyses of attitude in the association between knowledge and practice.

Model and path	B	SE	t value	*p* value	95% CI
Panel A. Unadjusted model					
Total-effect model					
Knowledge → Practice, path c	0.699	0.024	29.258	<0.001	0.652 to 0.746
Indirect-effect model					
Knowledge → Attitude, path a	0.328	0.024	13.456	<0.001	0.281 to 0.376
Attitude → Practice, path b	−0.059	0.043	−1.385	0.167	−0.143 to 0.025
Knowledge → Practice, path c′	0.718	0.028	25.930	<0.001	0.664 to 0.773
Knowledge → Attitude → Practice, a × b	−0.019	0.018	—	—	−0.056 to 0.015
Panel B. Covariate-adjusted model					
Total-effect model					
Knowledge → Practice, path c	0.291	0.032	9.047	<0.001	0.228 to 0.354
Indirect-effect model					
Knowledge → Attitude, path a	0.272	0.032	8.516	<0.001	0.209 to 0.335
Attitude → Practice, path b	0.048	0.044	1.073	0.284	−0.038 to 0.134
Knowledge → Practice, path c′	0.278	0.034	8.093	<0.001	0.211 to 0.345
Knowledge → Attitude → Practice, a × b	0.013	0.015	—	—	−0.015 to 0.044

B, unstandardized coefficient; SE, standard error; CI, confidence interval. The analyses were conducted using the Hayes PROCESS macro, Model 4. Knowledge, Attitude, and Practice were entered as continuous standardized scores on a 0–100 scale. Panel A shows the unadjusted exploratory model. Panel B shows the covariate-adjusted model, adjusted for residence, current pain intensity, age group, sex, and cancer stage. The indirect associations were estimated using bootstrapping with 5,000 resamples. For the indirect-effect rows, the 95% CI represents the percentile bootstrap confidence interval. For the indirect-effect rows, SE represents the bootstrap standard error, and no *t* value or *p* value is reported because inference was based on the percentile bootstrap 95% confidence interval. In both the unadjusted and covariate-adjusted models, the bootstrap confidence interval for the indirect effect included zero, indicating that the data did not support a statistically significant indirect association through Attitude. Because the data were cross-sectional, these results should be interpreted as exploratory descriptive decompositions of observed associations rather than evidence of temporal ordering or causal mediation. For regression path coefficients, 95% CIs were calculated from the corresponding standard errors.

In the unadjusted model, Knowledge was significantly associated with Practice in the total-effect model (path c: B = 0.699, SE = 0.024, *p* < 0.001) and was also significantly associated with Attitude (path a: B = 0.328, SE = 0.024, *p* < 0.001). However, Attitude was not significantly associated with Practice after Knowledge was included in the model (path b: B = −0.059, SE = 0.043, *p* = 0.167). The direct association between Knowledge and Practice remained statistically significant after Attitude was included (path c′: B = 0.718, SE = 0.028, *p* < 0.001). The bootstrap confidence interval for the unadjusted indirect effect included zero (indirect effect = −0.019; 95% bootstrap CI, −0.056 to 0.015).

In the covariate-adjusted model, Knowledge remained significantly associated with Practice in the total-effect model (path c: B = 0.291, SE = 0.032, *p* < 0.001) and was also significantly associated with Attitude (path a: B = 0.272, SE = 0.032, *p* < 0.001). However, Attitude was not significantly associated with Practice after Knowledge and covariates were included in the model (path b: B = 0.048, SE = 0.044, *p* = 0.284). The direct association between Knowledge and Practice remained statistically significant (path c′: B = 0.278, SE = 0.034, *p* < 0.001). The bootstrap confidence interval for the adjusted indirect effect also included zero (indirect effect = 0.013; 95% bootstrap CI, −0.015 to 0.044).

Therefore, neither the unadjusted nor the covariate-adjusted model supported a statistically significant indirect association through Attitude. These findings should be interpreted as exploratory and descriptive rather than as evidence of temporal ordering or causal mediation.

## 4 Discussion

This two-center cross-sectional study examined cancer pain-related knowledge, attitudes, and practices among patients with cancer-related pain in Southwest China and identified factors associated with pain-management practice. Overall, Knowledge and Attitude scores were moderate, whereas Practice scores were comparatively lower, suggesting a knowledge-to-action gap in cancer pain management in this region. In other words, the issue may not be limited to whether patients know about cancer pain management, but also whether this knowledge can be consistently translated into concrete behaviors, such as taking analgesics regularly, reporting pain in a timely manner, managing adverse effects appropriately, and seeking help when needed. This finding suggests that knowledge education alone may be insufficient to improve cancer pain-management practice, and that greater attention should be paid to behavioral implementation, clinical support, and service accessibility (29, 30).

In the multivariable analysis, higher Knowledge score and peri-urban or urban residence were associated with higher Practice scores, whereas greater current pain intensity, older age, and female sex were associated with lower Practice scores. Stage IV disease was associated with higher Practice scores after adjustment, although the unadjusted analysis showed lower Practice scores among patients with stage IV disease. This adjusted association remained generally consistent in the additional sensitivity model including education level. Nevertheless, this finding should be interpreted cautiously because the direction differed between the unadjusted and adjusted analyses, and residual confounding by center-level care patterns, disease-management intensity, palliative-care exposure, or other unmeasured clinical factors cannot be excluded.

Neither the unadjusted nor the covariate-adjusted exploratory indirect-effect analysis showed a statistically significant indirect association through Attitude. Although Knowledge was significantly associated with Attitude, Attitude did not statistically explain the association between Knowledge and Practice in the present model. Therefore, the knowledge-to-action pathway in cancer pain management may not follow a simple linear sequence of “knowledge → attitude → practice.” Behavior change theory emphasizes that health behavior depends on the joint contribution of capability, opportunity, and motivation (30). Multilevel intervention research also suggests that behavior change is embedded within broader interpersonal, organizational, and service-system contexts (31). More specifically, pain-management practice may be influenced by knowledge level, symptom burden, access to health services, and availability of analgesic medications (29, 32, 33). It may also be constrained by patient–clinician communication, follow-up support, caregiver involvement, and primary care capacity (31, 34, 35). Some of these factors require direct measurement in future studies.

The positive association between Knowledge and Practice is consistent with previous research. Prior studies suggest that pain-related knowledge, attitudinal barriers, analgesic adherence, and pain-management behaviors are related to cancer pain-management outcomes (11–14, 29). Patients with better understanding of pain reporting, regular analgesic use, misconceptions about opioids, adverse-effect management, and self-management principles may be more likely to report appropriate pain-management behaviors (11–14, 29). However, Practice scores in this study were lower than Knowledge and Attitude scores, indicating that knowledge itself does not necessarily translate into action. Even when patients understand the importance of cancer pain management, their behavior may still be affected by the physical and psychological burden of pain (8, 11), as well as concerns about opioid addiction, adverse effects, or stigma (32, 33). In addition, unclear use of rescue medication (36), insufficient follow-up, limited caregiver support (35), and inadequate patient–clinician communication (8, 11) may make it difficult for patients to sustain standardized pain-management behaviors over time. Future studies should therefore move beyond assessing what patients know and further identify the specific barriers that prevent knowledge from being translated into action (29–31).

The finding that Attitude did not statistically explain the Knowledge–Practice association deserves attention. The traditional KAP framework generally assumes that improved knowledge promotes attitude change, which in turn facilitates better practice (15). However, in the context of cancer pain management, behavior formation may depend on more complex clinical and social conditions (30, 31). Patients may recognize the importance of regular analgesic use and active pain reporting, but their actual behaviors may still be limited if medications are difficult to obtain, follow-up support is insufficient, pain suddenly worsens, adverse effects are feared, family assistance is lacking, or timely guidance from health professionals is unavailable (32, 33, 35). Therefore, Attitude remains an important component of the KAP framework, but in this study it was not the main intermediate link between Knowledge and Practice. Future research on cancer pain-management behavior may benefit from incorporating behavior change theory, implementation science, or multilevel intervention frameworks to examine the combined roles of individuals, families, patient–clinician communication, health-care institutions, and health systems (30, 31).

Residence was one of the factors most strongly associated with Practice scores. Compared with rural patients, peri-urban and urban patients had significantly higher Practice scores, indicating that urban–rural disparities remain important in cancer pain management in Southwest China. This finding is consistent with evidence on urban–rural differences in health-care utilization among patients with cancer in China and health inequities among rural residents in Southwest China (16, 17). Importantly, lower Practice scores among rural patients should not be simply attributed to insufficient individual knowledge or poor adherence, but should be understood in the context of health equity and regional health-service systems (17, 31). Rural patients may have less access to specialized pain-management services, standardized pain assessment, and continuous follow-up (16, 34). They may also have fewer opportunities to receive analgesic counseling, guidance on appropriate opioid use, and clear referral pathways (34). Because the present sample was recruited from two tertiary hospitals, the rural patients included in this study may already represent a subgroup with relatively better access to care. Therefore, the observed KAP levels may overestimate the true situation in the broader regional population, particularly among patients from remote rural areas, those with lower educational levels, and those with limited access to health services (17, 34). Improving cancer pain-management practice among rural patients requires not only patient education (29), but also stronger primary care capacity for pain assessment, standardized analgesic management, remote follow-up mechanisms, and optimized referral pathways (31, 34).

The relationship between pain intensity and Practice also requires careful interpretation. Current severe pain was associated with lower Practice scores, suggesting that patients experiencing severe pain may find it more difficult to maintain appropriate pain-management behaviors. Severe pain may impair physical functioning, sleep, mood, attention, and the ability to carry out treatment recommendations, thereby weakening patients’ ability to take medications regularly, record changes in pain, communicate actively, and manage adverse effects (37). Conversely, suboptimal pain-management practice may also be linked to inadequate pain control, such as failure to use baseline analgesics regularly, incorrect use of rescue medication, or delayed reporting of breakthrough pain (36). Patients may also reduce or stop medication because of concerns about adverse effects, opioid-related risks, or stigma (32, 33). Because this study was cross-sectional, and because current pain intensity and pain-management practice during the previous week were measured over different time frames, the direction of the relationship between pain intensity and practice cannot be determined (38). In addition, the association with moderate pain intensity was less stable: it was statistically significant in the ordinary least-squares model but not in the robust standard-error sensitivity analysis. Future studies should collect current pain, average pain, worst recent pain, breakthrough pain, pain relief, medication adjustment processes, and longitudinal pain trajectories to better clarify the relationship between pain experience and pain-management behavior (37, 38).

The findings related to cancer stage should also be interpreted cautiously. In the unadjusted analysis, patients with stage IV disease had lower Practice scores than those with stage I–III disease. However, after adjustment for Knowledge score, residence, current pain intensity, age, and sex, stage IV disease was associated with higher Practice scores. This reversal should not be interpreted as evidence that advanced cancer itself directly leads to better pain-management practice. A more plausible explanation is that cancer stage may not directly determine patients’ practice behaviors, but may be intertwined with frequency of medical contact, symptom-management needs, palliative care involvement, nursing education opportunities, and follow-up support (39–41). Some patients with stage IV disease may have a higher symptom burden, more frequent clinical visits, or earlier contact with oncology, pain medicine, nursing, and palliative care teams, thereby receiving more pain assessment, analgesic guidance, and self-management education (40, 41). Current oncology and palliative care guidelines also support earlier and more integrated palliative care for patients with advanced cancer to improve symptom control, quality of life, and care planning (39, 42, 43). However, this study did not directly measure service intensity, pain clinic consultation, palliative care referral, follow-up frequency, caregiver involvement, or family support. Therefore, the adjusted association with cancer stage should be regarded as hypothesis-generating and should be further examined in prospective studies.

The relationship between regular analgesic use and Practice also requires cautious interpretation. Regular analgesic use was not included in the primary regression model because of its conceptual overlap with medication-related behaviors in the Practice domain. In the sensitivity analysis, regular analgesic use was associated with higher Practice scores, and the direction of the main associations was generally unchanged. This may reflect better adherence to medical advice, greater engagement in pain management, or more communication with health professionals (44, 45), but it should not be interpreted as evidence that regular analgesic use independently causes better Practice scores (36). Previous studies have shown that analgesic adherence among patients with cancer pain is influenced by medication beliefs, concerns about analgesics, and treatment experiences (45, 46). In the context of opioid use, fear of addiction, stigma, and barriers to medication access may further affect actual medication-taking behavior (32, 33). Future research should further decompose “regular medication use” into more specific behavioral dimensions, including whether patients take baseline analgesics on schedule, use rescue medication appropriately, reduce or stop medication on their own, avoid medication because of concerns about addiction or adverse effects, actively report adverse reactions, and adjust doses under medical guidance (36). Such detailed assessment would help identify more specific behavioral barriers in cancer pain management (36).

Older age and female sex were also associated with lower Practice scores. These associations may reflect differences in individual factors, such as health literacy, comorbidity burden, functional limitations, and medication concerns, as well as external factors, such as caregiver support, patient–clinician communication, psychological distress, or social support. With respect to sex differences, previous research has suggested that female patients with cancer may experience greater pain burden, more hesitation about analgesic use, and lower analgesic adherence, indicating that sex-related differences may influence pain-management behavior and pain-control outcomes (47). Family caregivers’ knowledge, communication, and ability to provide assistance may also affect patients’ pain reporting, medication use, and self-management processes (35). Pain-management practice among older patients may be affected by functional status, medication complexity, dependence on caregivers, and insufficient ongoing medication education. However, these potential explanatory factors were not fully measured in the present study. Therefore, these subgroup findings should be viewed as clues for future research and intervention design rather than as definitive causal explanations. Future studies should further examine differences among older patients and female patients in pain expression, medication decision-making, reliance on family care, psychological burden, and medical communication, in order to develop more targeted support strategies (35, 47).

The present findings may inform cancer pain education and service planning in Southwest China. Cancer pain-management interventions should not remain limited to information delivery, but should move from “increasing knowledge” toward “facilitating knowledge-to-action translation” (29, 30). For patients with insufficient knowledge, basic cancer pain education should be strengthened, including pain reporting, regular analgesic use, opioid safety, adverse-effect management, rescue medication use, and communication with clinicians (4, 5, 29). For patients with medication concerns, opioid stigma, or reluctance to report pain, patient-centered communication should be used to address concerns about addiction, adverse effects, stigma, and the perceived need to endure pain (7, 11, 32, 33). For patients with adequate knowledge and attitudes but poor practice, interventions should focus on behavioral implementation, such as individualized medication plans, pain-recording tools, follow-up reminders, caregiver involvement, and support for practical difficulties in daily pain management (29, 35, 37). For rural patients, training in primary care pain assessment, remote education and follow-up, standardized analgesic supply, and clear two-way referral pathways should be strengthened (16, 34). For patients with severe pain or advanced disease, collaborative management by oncology, pain medicine, nursing, and palliative care teams should be prioritized rather than relying on one-time health education alone (39, 43). In other words, future strategies should not only increase what patients know, but also help patients engage in appropriate practices, perform them correctly, and sustain them in real-world health-care and family-care contexts.

These findings add patient-level KAP evidence on cancer pain management in Southwest China and situate inadequate practice within the broader context of regional service accessibility and health equity (16, 17, 34). Unlike studies that have mainly focused on the knowledge and practice of health-care providers, this study emphasizes patients’ own roles in pain reporting, medication use, and self-management (9, 10, 29, 36). In addition, by examining the indirect association among Knowledge, Attitude, and Practice, this study found that Attitude alone did not explain the knowledge-to-action gap, suggesting that future interventions should address both individual patient behavior and health-service support conditions (30, 31). Thus, the value of this study lies not only in describing the KAP status of patients with cancer pain in Southwest China, but also in indicating that quality improvement in cancer pain management should move beyond patient education alone toward behavioral support, family collaboration, and service-system optimization. Overall, this study provides evidence to inform the development of regionalized, behavior-oriented, and equity-focused cancer pain-management interventions (29–31, 34).

### 4.1 Limitations

This study has several limitations. First, the cross-sectional design precludes inference about temporal order or causality. Associations among Knowledge, Attitude, Practice, pain intensity, and cancer stage should not be interpreted as causal relationships (38). The exploratory indirect-effect analyses were also based on cross-sectional data and should therefore be understood only as descriptive decompositions of observed associations rather than evidence of causal mediation (20, 38). Although both unadjusted and covariate-adjusted models were performed and the bootstrap confidence intervals for the indirect effect through Attitude included zero in both models, this finding only indicates that Attitude did not statistically explain the Knowledge–Practice association in the present sample. It does not exclude the possibility of more complex pathways in other populations or longitudinal contexts. Future studies should use prespecified longitudinal models to examine whether dynamic temporal relationships exist among Knowledge, Attitude, and Practice (38).

Second, participants were recruited using convenience sampling from two tertiary hospitals in Southwest China, which limits the external validity of the findings. Patients treated in tertiary hospitals may have better access to oncology specialists, pain-management services, palliative care, health education, and analgesic prescriptions than patients treated in community hospitals, primary care clinics, rural health stations, or home-care settings. Therefore, the observed KAP scores may overestimate the true levels in the broader regional population, especially among patients from remote rural areas, those with lower educational levels, and those with limited access to health services (16, 17, 34). Future studies should include more regions, different levels of health-care institutions, and patients receiving community-based or home-based care to improve representativeness.

Third, this study used a self-developed questionnaire whose validation remains preliminary. Although the full questionnaire was provided and expert review, pilot testing, content validity assessment, internal consistency analysis, item-level evaluation, and confirmatory factor analysis were performed, test–retest reliability, criterion validity, convergent validity, discriminant validity, responsiveness, measurement invariance, and validation in an independent sample have not yet been assessed (22, 26, 27, 48). Therefore, the questionnaire should not be considered a fully validated measurement instrument. In addition, because the questionnaire included both dichotomously scored Knowledge items and Likert-type Attitude and Practice items, the confirmatory factor analysis results should be interpreted cautiously as preliminary structural evidence rather than definitive validation evidence (27, 48). The English version of the questionnaire was provided only for publication reference and was not independently validated. Future studies should further validate this instrument in independent multicenter samples and use methods more appropriate for categorical or ordinal data, such as confirmatory factor analysis based on tetrachoric or polychoric correlations with robust weighted least-squares estimation (27, 48).

Fourth, Practice behaviors were self-reported and may have been affected by recall bias, social desirability bias, and common method bias, particularly because most data were collected through face-to-face interviews (49). Notably, the main regression model explained a high proportion of variance in Practice scores (R^2^ = 0.789). Although this may partly reflect a true association between Knowledge and Practice, common method variance may have inflated the associations because Knowledge, Attitude, and Practice were measured using the same self-report questionnaire, at the same time point, and in a similar survey context (49). Therefore, the high explanatory power of the model should be interpreted cautiously. The model is better understood as identifying explanatory associations within the present dataset rather than as a prediction model with established external validity. Future research could incorporate objective indicators, such as prescription records, medication logs, follow-up records, pain diaries, or electronic symptom-monitoring data, to reduce bias from reliance on a single self-report source (37, 49).

Fifth, current pain intensity was assessed at the time of the survey, whereas Practice items referred to behaviors during the previous week. Therefore, the temporal relationship between pain experience and practice behavior could not be determined (38). Pain intensity may influence patients’ ability to perform pain-management behaviors, but it may also be affected by prior pain-management behaviors. Future research should collect current pain, average pain, worst recent pain, breakthrough pain, pain relief, and longitudinal pain changes to more accurately examine the relationship between pain burden and practice behavior (37, 38).

Sixth, although this study collected a broad range of cancer diagnosis categories and several sociodemographic variables, it did not fully capture detailed tumor-related, treatment-related, socioeconomic, and health-service factors. Specific histological subtype, metastatic site, current anticancer treatment, analgesic regimen, opioid dose, breakthrough-pain medication, ECOG performance status, psychological distress, income, health insurance, travel distance to care, caregiver involvement, center-level differences, and health-care providers’ educational practices were not sufficiently measured to support reliable adjustment (4, 5, 32, 33, 35, 36). Education level was not included in the primary multivariable model but was examined in an additional sensitivity model, in which the main findings remained generally unchanged. Ethnicity and pain duration were examined in descriptive and univariate analyses but were not retained in the primary multivariable model. Recruitment center could not be included in the regression models because no participant-level recruitment-center variable was available for analysis in the final de-identified analytic dataset. These unmeasured, insufficiently measured, or unmodeled factors may have led to residual confounding, especially by center-level care patterns, disease-management intensity, palliative-care exposure, or other health-service factors; therefore, the observed associations should be interpreted cautiously (38).

Finally, regular analgesic use was examined only in the sensitivity analysis because it overlapped conceptually with medication-related behaviors in the Practice domain. Therefore, findings related to this variable should be interpreted with caution (36, 44–46). The sample-size estimation was based primarily on the planned regression model, and subgroup comparisons, confirmatory factor analysis, and exploratory indirect-effect analyses should be considered supportive or hypothesis-generating rather than confirmatory analyses with independent adequate power.

## 5 Conclusion

In this two-center cross-sectional study of patients with cancer-related pain in Southwest China, Knowledge and Attitude scores were moderate, whereas Practice scores were comparatively lower, suggesting an apparent knowledge-to-action gap in cancer pain management. Higher Knowledge score and peri-urban or urban residence were associated with better Practice, whereas greater current pain intensity, older age, and female sex were associated with lower Practice scores. Stage IV disease was positively associated with Practice after multivariable adjustment, and this finding remained generally consistent in the additional sensitivity model including education level; however, it should be interpreted cautiously because unadjusted analyses showed the opposite direction. Neither the unadjusted nor the covariate-adjusted exploratory indirect-effect analysis supported a statistically significant indirect association through Attitude, indicating that the Knowledge–Practice association observed in this sample was not statistically explained by Attitude alone. These findings suggest that improving cancer pain-management practice may require more than increasing patients’ knowledge or improving attitudes; greater attention should be given to whether patients are able to translate pain-management knowledge into sustained daily behaviors, particularly in relation to analgesic use, timely pain reporting, communication with clinicians, and access to follow-up support. Future prospective, multicenter, and community-based studies are needed to validate the questionnaire, clarify the temporal relationships among Knowledge, Attitude, and Practice, and develop context-sensitive, equity-focused, and behavior-oriented strategies for cancer pain management.

## Data Availability

The original contributions presented in the study are included in the article/Supplementary material, further inquiries can be directed to the corresponding authors.
