# Supplementary material for: Knowledge, attitude, and practice regarding cancer pain management in Southwest China: a two-center cross-sectional study
Source: Front Public Health. 2026 Jul 22;14:1871970. doi: 10.3389/fpubh.2026.1871970 (PMC13440342; doi:10.3389/fpubh.2026.1871970)
Supplement: Supplementary file 1 [file Data_Sheet_1.docx]

Supplementary Material 1. Item-Level Analyses and Preliminary Psychometric Properties of the Cancer Pain Management KAP Questionnaire

The full cancer pain Knowledge, Attitude, and Practice (KAP) questionnaire is provided in Supplementary Material 3, including the original Chinese version used for data collection and an English translation for publication reference. The present supplementary material reports item-level distributions, corrected item-total correlations, internal consistency indices, reliability coefficients if item deleted, and floor/ceiling effects based on the final scored analytic dataset. These results are intended to improve transparency regarding item performance and preliminary psychometric properties in the present sample.

The values reported in this supplementary file are based on the final scored analytic dataset rather than unprocessed raw responses. Reverse-worded items included A2, A3, A4, A6, and P8. These items were reverse-coded according to the predefined scoring scheme before any item-level, subscale-level, or subsequent statistical analyses were conducted. Therefore, higher scored values consistently indicate greater knowledge, more positive attitudes, or more appropriate pain-management practices.

Because the questionnaire included mixed dichotomous and ordinal item formats, and because the CFA was interpreted only as preliminary structural evidence, item-level CFA factor loadings are not presented as definitive validation evidence. These results should be interpreted as preliminary psychometric evidence regarding item performance and internal consistency in the present sample, rather than as full validation of the instrument. Further studies are needed to evaluate test-retest reliability, criterion validity, convergent and discriminant validity, measurement invariance, responsiveness, and validation in independent samples.

**Supplementary Table S1. Knowledge scored item distribution (N = 523)**

| **Item** | **Correct/scored 1 n (%)** | **Incorrect or unclear/scored 0 n (%)** |
| --- | --- | --- |
| K1 | 385 (73.6%) | 138 (26.4%) |
| K2 | 366 (70.0%) | 157 (30.0%) |
| K3 | 353 (67.5%) | 170 (32.5%) |
| K4 | 247 (47.2%) | 276 (52.8%) |
| K5 | 146 (27.9%) | 377 (72.1%) |
| K6 | 366 (70.0%) | 157 (30.0%) |
| K7 | 313 (59.8%) | 210 (40.2%) |
| K8 | 390 (74.6%) | 133 (25.4%) |

**Note.** Knowledge items were dichotomously scored, with 1 indicating a correct answer and 0 indicating an incorrect or unclear answer.

**Supplementary Table S2. Attitude scored item descriptive statistics (N = 523)**

| **Item** | **Mean ± SD** | **Median (IQR)** |
| --- | --- | --- |
| A1 | 3.82 ± 1.35 | 4.0 (3.0–5.0) |
| A2* | 2.86 ± 1.39 | 3.0 (2.0–4.0) |
| A3* | 2.67 ± 1.47 | 2.0 (1.0–4.0) |
| A4* | 2.53 ± 1.55 | 2.0 (1.0–4.0) |
| A5 | 4.08 ± 1.07 | 4.0 (4.0–5.0) |
| A6* | 2.61 ± 1.47 | 2.0 (1.0–4.0) |
| A7 | 3.96 ± 1.16 | 4.0 (3.0–5.0) |
| A8 | 3.86 ± 1.35 | 4.0 (3.0–5.0) |

**Note.** Values are based on the final scored/coded analytic dataset. A2, A3, A4, and A6 were reverse–worded items and had already been reverse–coded before these statistics were generated. Higher values indicate more positive attitudes.

**Supplementary Table S3. Attitude scored item category distribution (N = 523)**

| **Item** | **Scored category 1 n (%)** | **Scored category 2 n (%)** | **Scored category 3 n (%)** | **Scored category 4 n (%)** | **Scored category 5 n (%)** |
| --- | --- | --- | --- | --- | --- |
| A1 | 52 (9.9%) | 52 (9.9%) | 64 (12.2%) | 123 (23.5%) | 232 (44.4%) |
| A2* | 94 (18.0%) | 158 (30.2%) | 102 (19.5%) | 64 (12.2%) | 105 (20.1%) |
| A3* | 135 (25.8%) | 164 (31.4%) | 70 (13.4%) | 44 (8.4%) | 110 (21.0%) |
| A4* | 173 (33.1%) | 166 (31.7%) | 38 (7.3%) | 25 (4.8%) | 121 (23.1%) |
| A5 | 11 (2.1%) | 53 (10.1%) | 53 (10.1%) | 173 (33.1%) | 233 (44.6%) |
| A6* | 147 (28.1%) | 156 (29.8%) | 79 (15.1%) | 36 (6.9%) | 105 (20.1%) |
| A7 | 11 (2.1%) | 71 (13.6%) | 86 (16.4%) | 117 (22.4%) | 238 (45.5%) |
| A8 | 52 (9.9%) | 52 (9.9%) | 54 (10.3%) | 126 (24.1%) | 239 (45.7%) |

**Note.** The category distributions are based on final scored/coded item values. For reverse–worded items (A2, A3, A4, and A6), categories reflect values after reverse–coding, not unprocessed raw responses.

**Supplementary Table S4. Practice scored item descriptive statistics (N = 523)**

| **Item** | **Mean ± SD** | **Median (IQR)** |
| --- | --- | --- |
| P1 | 3.42 ± 1.20 | 4.0 (3.0–4.0) |
| P2 | 3.37 ± 1.46 | 4.0 (3.0–4.0) |
| P3 | 3.18 ± 1.23 | 3.0 (2.0–4.0) |
| P4 | 1.94 ± 1.04 | 2.0 (1.0–3.0) |
| P5 | 3.36 ± 1.18 | 4.0 (3.0–4.0) |
| P6 | 2.70 ± 1.21 | 2.0 (2.0–4.0) |
| P7 | 3.64 ± 1.10 | 4.0 (3.0–5.0) |
| P8* | 2.63 ± 1.43 | 2.0 (1.0–4.0) |

**Note.** Values are based on the final scored/coded analytic dataset. P8 was a reverse–worded item and had already been reverse–coded before these statistics were generated. Higher values indicate more appropriate pain–management practices.

**Supplementary Table S5. Practice scored item category distribution (N = 523)**

| **Item** | **Scored category 1 n (%)** | **Scored category 2 n (%)** | **Scored category 3 n (%)** | **Scored category 4 n (%)** | **Scored category 5 n (%)** |
| --- | --- | --- | --- | --- | --- |
| P1 | 63 (12.0%) | 46 (8.8%) | 101 (19.3%) | 235 (44.9%) | 78 (14.9%) |
| P2 | 122 (23.3%) | 2 (0.4%) | 83 (15.9%) | 191 (36.5%) | 125 (23.9%) |
| P3 | 64 (12.2%) | 79 (15.1%) | 162 (31.0%) | 134 (25.6%) | 84 (16.1%) |
| P4 | 225 (43.0%) | 155 (29.6%) | 112 (21.4%) | 10 (1.9%) | 21 (4.0%) |
| P5 | 53 (10.1%) | 71 (13.6%) | 108 (20.7%) | 217 (41.5%) | 74 (14.1%) |
| P6 | 88 (16.8%) | 178 (34.0%) | 106 (20.3%) | 106 (20.3%) | 45 (8.6%) |
| P7 | 13 (2.5%) | 78 (14.9%) | 133 (25.4%) | 158 (30.2%) | 141 (27.0%) |
| P8* | 142 (27.2%) | 147 (28.1%) | 96 (18.4%) | 40 (7.6%) | 98 (18.7%) |

**Note.** The category distributions are based on final scored/coded item values. For the reverse–worded item P8, categories reflect values after reverse–coding, not unprocessed raw responses.

**Supplementary Table S6. Corrected item–total correlations and internal consistency coefficients (N = 523)**

| **Item/subscale** | **Mean** | **SD** | **CITC** | **Reliability coefficient if item deleted** |
| --- | --- | --- | --- | --- |
| **Knowledge (KR–20)** |  |  |  |  |
| K1 | 0.7361 | 0.4411 | 0.6641 | 0.8284 |
| K2 | 0.6998 | 0.4588 | 0.4583 | 0.8519 |
| K3 | 0.6750 | 0.4688 | 0.7059 | 0.8226 |
| K4 | 0.4723 | 0.4997 | 0.6868 | 0.8245 |
| K5 | 0.2792 | 0.4490 | 0.4309 | 0.8546 |
| K6 | 0.6998 | 0.4588 | 0.6935 | 0.8244 |
| K7 | 0.5985 | 0.4907 | 0.4997 | 0.8482 |
| K8 | 0.7457 | 0.4359 | 0.6350 | 0.8319 |
| **Scale KR–20** | **0.8538** |  |  |  |
| **Attitude (Cronbach alpha)** |  |  |  |  |
| A1 | 3.8241 | 1.3532 | 0.8815 | 0.9473 |
| A2* | 2.8623 | 1.3897 | 0.8774 | 0.9475 |
| A3* | 2.6750 | 1.4731 | 0.8352 | 0.9504 |
| A4* | 2.5315 | 1.5488 | 0.7730 | 0.9551 |
| A5 | 4.0784 | 1.0665 | 0.8697 | 0.9500 |
| A6* | 2.6099 | 1.4651 | 0.7644 | 0.9550 |
| A7 | 3.9560 | 1.1630 | 0.8734 | 0.9488 |
| A8 | 3.8566 | 1.3549 | 0.8726 | 0.9478 |
| **Scale alpha** | **0.9562** |  |  |  |
| **Practice (Cronbach alpha)** |  |  |  |  |
| P1 | 3.4187 | 1.2013 | 0.8953 | 0.9743 |
| P2 | 3.3728 | 1.4571 | 0.8958 | 0.9753 |
| P3 | 3.1816 | 1.2284 | 0.9404 | 0.9719 |
| P4 | 1.9426 | 1.0397 | 0.8185 | 0.9782 |
| P5 | 3.3595 | 1.1813 | 0.9473 | 0.9717 |
| P6 | 2.6979 | 1.2132 | 0.9148 | 0.9733 |
| P7 | 3.6424 | 1.1039 | 0.9539 | 0.9718 |
| P8* | 2.6272 | 1.4346 | 0.9274 | 0.9732 |
| **Scale alpha** | **0.9770** |  |  |  |

**Note.** CITC = corrected item–total correlation. All results in this table are based on the final scored/coded analytic dataset. Reverse-worded items (A2, A3, A4, A6, and P8) had already been reverse-coded before CITC and internal consistency coefficients were calculated. The KR–20 coefficient was used for the dichotomously scored Knowledge subscale, whereas Cronbach alpha was used for the Likert-type Attitude and Practice subscales. An overall 24-item internal consistency coefficient was not interpreted as a primary reliability index because the scale combines dichotomously scored Knowledge items and Likert-type Attitude and Practice items; subscale-specific coefficients should be interpreted as the primary reliability indices.

**Supplementary Table S7. Floor and ceiling effects based on scored/coded item values (N = 523)**

| **Item/subscale** | **Floor n (%)** | **Ceiling n (%)** | **Floor effect** | **Ceiling effect** |
| --- | --- | --- | --- | --- |
| K1 | 138 (26.4%) | 385 (73.6%) | Yes | Yes |
| K2 | 157 (30.0%) | 366 (70.0%) | Yes | Yes |
| K3 | 170 (32.5%) | 353 (67.5%) | Yes | Yes |
| K4 | 276 (52.8%) | 247 (47.2%) | Yes | Yes |
| K5 | 377 (72.1%) | 146 (27.9%) | Yes | Yes |
| K6 | 157 (30.0%) | 366 (70.0%) | Yes | Yes |
| K7 | 210 (40.2%) | 313 (59.8%) | Yes | Yes |
| K8 | 133 (25.4%) | 390 (74.6%) | Yes | Yes |
| A1 | 52 (9.9%) | 232 (44.4%) | No | Yes |
| A2* | 94 (18.0%) | 105 (20.1%) | Yes | Yes |
| A3* | 135 (25.8%) | 110 (21.0%) | Yes | Yes |
| A4* | 173 (33.1%) | 121 (23.1%) | Yes | Yes |
| A5 | 11 (2.1%) | 233 (44.6%) | No | Yes |
| A6* | 147 (28.1%) | 105 (20.1%) | Yes | Yes |
| A7 | 11 (2.1%) | 238 (45.5%) | No | Yes |
| A8 | 52 (9.9%) | 239 (45.7%) | No | Yes |
| P1 | 63 (12.0%) | 78 (14.9%) | No | No |
| P2 | 122 (23.3%) | 125 (23.9%) | Yes | Yes |
| P3 | 64 (12.2%) | 84 (16.1%) | No | Yes |
| P4 | 225 (43.0%) | 21 (4.0%) | Yes | No |
| P5 | 53 (10.1%) | 74 (14.1%) | No | No |
| P6 | 88 (16.8%) | 45 (8.6%) | Yes | No |
| P7 | 13 (2.5%) | 141 (27.0%) | No | Yes |
| P8* | 142 (27.2%) | 98 (18.7%) | Yes | Yes |
| Knowledge total score (0–8) | 47 (9.0%) | 91 (17.4%) | No | Yes |
| Attitude total score (8–40) | 11 (2.1%) | 105 (20.1%) | No | Yes |
| Practice total score (8–40) | 11 (2.1%) | 4 (0.8%) | No | No |

**Note.** A threshold of ≥15% was used to indicate a potential floor or ceiling effect. All values were calculated from the final scored analytic dataset. For Knowledge items, floor and ceiling percentages represent the proportions of incorrect/unclear and correct responses, respectively. Because Knowledge items were dichotomously scored, floor and ceiling effects for these items should be interpreted cautiously and are mainly descriptive. For Attitude and Practice items, floor and ceiling percentages represent the proportions of responses in the lowest scored category and highest scored category, respectively. Reverse–worded items had already been reverse–coded before floor and ceiling effects were summarized.
*Reverse–worded item; values are shown after reverse–coding.
