# Supplementary material for: Knowledge, attitude, and practice regarding cancer pain management in Southwest China: a two-center cross-sectional study
Source: Front Public Health. 2026 Jul 22;14:1871970. doi: 10.3389/fpubh.2026.1871970 (PMC13440342; doi:10.3389/fpubh.2026.1871970)
Supplement: Supplementary file 2 [file Data_Sheet_2.docx]

**Supplementary Material 2. Regression Diagnostics and Sensitivity Analyses**

This supplementary material presents sensitivity analyses and diagnostic information for the revised multivariable regression model with Practice score (0–100) as the dependent variable. Regular analgesic use was evaluated in a sensitivity model because of its potential conceptual overlap with medication-related behaviors included in the Practice dimension. An additional sensitivity model including education level was also conducted to assess the robustness of the primary findings.

**Supplementary Table S8. Sensitivity Analysis Adding Regular Analgesic Use to the Primary Model**

| **Variable** | **Model A: primary model B (95% CI; P value)** | **Model B: + regular analgesic use B (95% CI; P value)** |
| --- | --- | --- |
| Knowledge score | 0.29 (0.23 to 0.35; <0.001) | 0.12 (0.04 to 0.20; 0.002) |
| Peri-urban residence (ref: rural) | 20.14 (15.67 to 24.61; <0.001) | 18.94 (14.61 to 23.27; <0.001) |
| Urban residence (ref: rural) | 28.56 (24.77 to 32.36; <0.001) | 28.52 (24.85 to 32.19; <0.001) |
| Moderate pain (ref: mild) | -7.95 (-13.62 to -2.28; 0.006) | -16.91 (-22.67 to -11.15; <0.001) |
| Severe pain (ref: mild) | -19.15 (-25.89 to -12.41; <0.001) | -30.50 (-37.37 to -23.63; <0.001) |
| Age ≥60 years (ref: <60 years) | -9.16 (-13.23 to -5.08; <0.001) | -8.23 (-12.33 to -4.13; <0.001) |
| Stage IV disease (ref: stage I–III) | 13.47 (7.64 to 19.29; <0.001) | 17.17 (11.83 to 22.51; <0.001) |
| Female sex (ref: male) | -4.64 (-7.28 to -2.00; <0.001) | -6.58 (-9.23 to -3.93; <0.001) |
| Regular analgesic use (ref: no) | Not included | 14.60 (10.01 to 19.19; <0.001) |

**Note.** Model A corresponds to the primary model reported in Table 5 (R² = 0.789; adjusted R² = 0.785). Model B added regular analgesic use to the primary model (R² = 0.803; adjusted R² = 0.799). Confidence intervals were calculated using ordinary least-squares standard errors, consistent with the primary regression model. The directions of the core associations remained unchanged after adding regular analgesic use, although the magnitude of some coefficients changed. Regular analgesic use was not retained in the primary model because of potential conceptual overlap with medication-related behaviors included in the Practice dimension.

**Supplementary Table S9. Robust Standard-Error Sensitivity Analysis for the Primary Regression Model**

| **Variable** | **B** | **Robust SE** | **95% CI** | **P value** |
| --- | --- | --- | --- | --- |
| Knowledge score | 0.29 | 0.05 | 0.20 to 0.38 | <0.001 |
| Peri-urban residence (ref: rural) | 20.14 | 2.41 | 15.41 to 24.87 | <0.001 |
| Urban residence (ref: rural) | 28.56 | 2.72 | 23.22 to 33.91 | <0.001 |
| Moderate pain (ref: mild) | -7.95 | 4.93 | -17.63 to 1.74 | 0.108 |
| Severe pain (ref: mild) | -19.15 | 4.90 | -28.77 to -9.52 | <0.001 |
| Age ≥60 years (ref: <60 years) | -9.16 | 2.02 | -13.12 to -5.19 | <0.001 |
| Stage IV disease (ref: stage I–III) | 13.47 | 3.81 | 5.98 to 20.95 | <0.001 |
| Female sex (ref: male) | -4.64 | 1.20 | -7.00 to -2.28 | <0.001 |

**Note.** This sensitivity analysis used heteroscedasticity-consistent robust standard errors. The directions of the main associations were generally consistent with the primary ordinary least-squares model, except that moderate current pain intensity was no longer statistically significant.

**Supplementary Table S10. Unadjusted Comparison of Practice Scores by Cancer Stage**

| **Cancer stage** | **n** | **Practice score, mean ± SD** | **Mean difference (Stage IV minus stage I–III)** | **t value** | **P value** |
| --- | --- | --- | --- | --- | --- |
| Stage I–III | 105 | 60.74 ± 24.63 | Reference | — | — |
| Stage IV | 418 | 51.42 ± 29.58 | -9.32 | -2.980 | 0.003 |

**Note.** Practice scores were compared between patients with stage I–III disease and those with stage IV disease without covariate adjustment. Patients with stage IV disease had lower unadjusted Practice scores than those with stage I–III disease. This analysis was used to describe the unadjusted association between cancer stage and Practice score before multivariable adjustment.

**Supplementary Table S11. Simple Linear Regression of Practice Score on Stage IV Disease**

| **Variable** | **B** | **SE** | **95% CI** | **Standardized β** | **t value** | **P value** | **VIF** | **Tolerance** |
| --- | --- | --- | --- | --- | --- | --- | --- | --- |
| Intercept | 60.744 | 2.797 | 55.25 to 66.24 | — | 21.718 | <0.001 | — | — |
| Stage IV disease | -9.324 | 3.129 | -15.47 to -3.18 | -0.129 | -2.980 | 0.003 | 1.000 | 1.000 |

**Note.** Stage IV disease was coded as 0 = stage I–III and 1 = stage IV according to the final analytic dataset. No covariates were included in this simple linear regression model. Model R² = 0.017; adjusted R² = 0.015; F(1, 521) = 8.881, P = 0.003; N = 523.

**Supplementary Table S12. Variable coding used in the statistical analyses**

| **Variable** | **Coding or treatment in analysis** |
| --- | --- |
| Knowledge score | Continuous standardized score, ranging from 0 to 100. |
| Attitude score | Continuous standardized score, ranging from 0 to 100. |
| Practice score | Continuous standardized score, ranging from 0 to 100; used as the dependent variable in the primary multivariable regression model. |
| Age group | Dichotomized as <60 years and ≥60 years. The <60 years group was used as the reference category, and the ≥60 years group was entered as the comparison category. |
| Sex | Male was used as the reference category, and female was entered as the comparison category. |
| Residence | Rural residence was used as the reference category. Peri-urban residence and urban residence were entered as dummy variables. |
| Current pain intensity | Categorized according to the Numeric Rating Scale (NRS): mild pain, NRS 1–3; moderate pain, NRS 4–6; and severe pain, NRS 7–10. Mild pain was used as the reference category, and moderate and severe pain were entered as dummy variables. |
| Cancer stage | Cancer stage was initially collected using broad questionnaire categories and subsequently coded in the final analytic dataset as stage I–III versus stage IV. Stage I–III was used as the reference category, and stage IV was entered as the comparison category. |
| Regular analgesic use | Categorized as no and yes. No regular analgesic use was used as the reference category, and regular analgesic use was entered as the comparison category. This variable was included only in the sensitivity model because of its potential conceptual overlap with medication-related behaviors included in the Practice domain. |
| Education level | Education level was categorized as low, intermediate, and high education. Low education was used as the reference category, and intermediate and high education were entered as separate dummy variables in the additional education-adjusted sensitivity model. |
| Ethnicity | Categorized as Han and ethnic minority. Ethnicity was used in descriptive and univariate analyses but was not retained in the primary multivariable regression model. |
| Pain duration | Categorized as <1 month, 1–3 months, 3–6 months, and >6 months. Pain duration was used in descriptive and univariate analyses but was not retained in the primary multivariable regression model. |
| Recruitment center | Recruitment center was used to describe the two-center study setting and recruitment process. No participant-level recruitment-center variable was available for analysis in the final de-identified analytic dataset; therefore, recruitment center could not be included in the primary or sensitivity regression models. |

**Note.** NRS, Numeric Rating Scale. Categorical variables in the primary multivariable regression model were entered using dummy coding, with the reference categories shown above. Regular analgesic use was evaluated only in a sensitivity model because it may overlap conceptually with medication-related behaviors included in the Practice domain.

Supplementary Table S13. Additional sensitivity model including education level

| **Variable** | **B** | **SE** | **95% CI** | **Standardized β** | **P value** | **VIF** |
| --- | --- | --- | --- | --- | --- | --- |
| Intercept | 30.12 | 2.43 | 25.35 to 34.89 | — | <0.001 | — |
| Knowledge score | 0.29 | 0.03 | 0.23 to 0.35 | 0.328 | <0.001 | 3.26 |
| Peri-urban residence vs rural | 20.54 | 2.71 | 15.22 to 25.87 | 0.285 | <0.001 | 3.44 |
| Urban residence vs rural | 29.18 | 3.59 | 22.12 to 36.24 | 0.496 | <0.001 | 9.03 |
| Moderate pain vs mild | -7.97 | 2.96 | -13.78 to -2.16 | -0.138 | 0.007 | 6.31 |
| Severe pain vs mild | -18.87 | 3.50 | -25.75 to -11.98 | -0.323 | <0.001 | 8.72 |
| Age ≥60 vs <60 years | -9.10 | 2.12 | -13.26 to -4.95 | -0.142 | <0.001 | 2.64 |
| Stage IV vs stage I–III | 13.07 | 3.01 | 7.16 to 18.98 | 0.182 | <0.001 | 4.23 |
| Female vs male | -4.60 | 1.58 | -7.69 to -1.50 | -0.079 | 0.004 | 1.78 |
| Intermediate education vs low | -0.71 | 3.09 | -6.77 to 5.36 | -0.012 | 0.819 | 6.85 |
| High education vs low | 1.49 | 3.85 | -6.08 to 9.06 | 0.012 | 0.699 | 2.34 |

**Note.** The dependent variable was standardized Practice score. This model added education level to the primary multivariable model. Low education was used as the reference category. Model R² = 0.789; adjusted R² = 0.785; F(10, 512) = 191.327, P < 0.001. Recruitment center could not be included because no participant-level recruitment-center variable was available for analysis in the final de-identified analytic dataset.

**Supplementary Figure S1. Distribution of Practice Scores.**
This figure shows the distribution of the standardized Practice score, which ranged from 0 to 100.

**Supplementary Figure S2. Normal Q–Q Plot of Regression Residuals.**
This figure was used to assess the normality of residuals in the primary multivariable linear regression model.

**Supplementary Figure S3. Standardized Residuals Versus Predicted Values.**
This figure was used to assess heteroscedasticity and model fit.

Note. The diagnostic plots suggested some deviation from normality; therefore, robust standard-error sensitivity analysis was conducted.
