# Supplementary material for: Knowledge, attitude, and practice regarding cancer pain management in Southwest China: a two-center cross-sectional study
Source: Front Public Health. 2026 Jul 22;14:1871970. doi: 10.3389/fpubh.2026.1871970 (PMC13440342; doi:10.3389/fpubh.2026.1871970)
Supplement: Supplementary file 3 [file Data_Sheet_3.docx]

**Supplementary Material 3. Cancer Pain Knowledge, Attitude, and Practice Questionnaire: Original Chinese Version and English Translation**

Note: The original Chinese version of this questionnaire was used for data collection in the present study. The English version is provided as a translation for publication and reader reference only and has not been independently validated as a separate English-language instrument. Reverse-scored items are marked with “(R)”. The scoring instructions shown in this supplementary file are provided for research transparency.

《西南地区癌痛知识-态度-行为（KAP）调查问卷》

尊敬的患者朋友，您好！

我们是昆明理工大学附属安宁市第一人民医院的研究团队，正在进行一项关于癌症疼痛管理的调查研究，希望能了解您对癌痛的看法和做法。本问卷为匿名填写，所有信息都将严格保密，且仅用于科学研究。您的真实回答对我们至关重要，完成问卷大约需要10-15分钟。

非常感谢您的支持与参与！

第一部分：您的基本情况 (请在相应选项上打“√”或填写)

您的年龄： ______岁

您的性别： □ 男 □ 女

您的民族： □ 汉族 □ 藏族 □ 彝族 □ 苗族 □ 其他 ______

您的文化程度： □ 小学及以下 □ 初中 □ 高中/中专 □ 大专及以上

您的居住地： □ 城市 □ 乡镇 □ 农村

您的癌症诊断： □ 肺癌 □ 肝癌 □ 胃肠道癌（食管、胃、结直肠） □ 乳腺癌 □ 妇科肿瘤 □ 其他 _________

您的癌症分期： □ 早期（I-II期） □ 晚期（III-IV期）/已发生转移 □ 不清楚

您现在最疼的地方有多疼？ (0分代表完全不痛，10分代表能想象到的最剧烈的疼痛)
0 -- 1 -- 2 -- 3 -- 4 -- 5 -- 6 -- 7 -- 8 -- 9 -- 10 (请圈出最符合的数字)

您出现这种癌症疼痛有多长时间了？
□ 少于1个月 □ 1-3个月 □ 3-6个月 □ 超过6个月

您目前是否正在规律服用止痛药？
□ 是 □ 否

第二部分：癌痛知识 (请选出您认为最合适的答案)

| 编号 | 问题 | 您的选择 |
| --- | --- | --- |
| K1 | 癌症的疼痛是可以被有效控制的。 | □ 是 □ 否 □ 不清楚 |
| K2 | 即使平时不痛，也可能突然出现短暂的剧烈疼痛（爆发痛）。 | □ 是 □ 否 □ 不清楚 |
| K3 | 按时服用止痛药比等痛得厉害再吃效果更好。 | □ 是 □ 否 □ 不清楚 |
| K4 | 医生会根据我的疼痛情况，慢慢帮我找到最合适的药量。 | □ 是 □ 否 □ 不清楚 |
| K5 | 吗啡等强止痛药只要在医生指导下使用，就不容易上瘾。 | □ 是 □ 否 □ 不清楚 |
| K6 | 止痛药的副作用（如便秘、恶心）是可以预防和处理的。 | □ 是 □ 否 □ 不清楚 |
| K7 | 除了吃药，听音乐、按摩、放松等方法也能帮助缓解疼痛。 | □ 是 □ 否 □ 不清楚 |
| K8 | 疼痛评分是帮助医生了解我有多痛的重要方法。 | □ 是 □ 否 □ 不清楚 |
| 计分方式（供研究者参考）答对计1分，答错或不清楚计0分。总分越高，知识水平越好。 | | |

第三部分：癌痛态度 (请根据您的真实想法，圈出最符合的数字)

评分标准：1=完全不同意, 2=不同意, 3=不确定, 4=同意, 5=完全同意

| 编号 | 观点 | 您的看法 |
| --- | --- | --- |
| A1 | 我相信通过治疗，我的疼痛可以得到很好的缓解。 | 1 -- 2 -- 3 -- 4 -- 5 |
| A2 | 忍受疼痛是意志坚强的表现。 (R) | 1 -- 2 -- 3 -- 4 -- 5 |
| A3 | 经常向医生说痛，会给医生和家人添麻烦。 (R) | 1 -- 2 -- 3 -- 4 -- 5 |
| A4 | 我非常担心吃吗啡这样的强止痛药会像吸毒一样上瘾。 (R) | 1 -- 2 -- 3 -- 4 -- 5 |
| A5 | 我觉得告诉医生我有多痛，对治疗很重要。 | 1 -- 2 -- 3 -- 4 -- 5 |
| A6 | 我担心止痛药的副作用比疼痛本身更难受。 (R) | 1 -- 2 -- 3 -- 4 -- 5 |
| A7 | 只要能止痛，即使有点副作用我也愿意接受。 | 1 -- 2 -- 3 -- 4 -- 5 |
| A8 | 只要医生开了止痛药，我就会积极配合使用。 | 1 -- 2 -- 3 -- 4 -- 5 |
| 计分方式（供研究者参考）正向条目1-5分计，反向条目(R)反向计分(5-1分)。总分越高，态度越积极。 | | |

第四部分：癌痛行为 (请根据您过去一周的实际情况，圈出最符合的数字)

评分标准：1=从不, 2=偶尔, 3=有时, 4=经常, 5=总是

| 编号 | 做法 | 您的频率 |
| --- | --- | --- |
| P1 | 我会主动告诉医生或护士我的疼痛程度和变化。 | 1 -- 2 -- 3 -- 4 -- 5 |
| P2 | 我会严格按照医生告诉我的时间和剂量吃止痛药。 | 1 -- 2 -- 3 -- 4 -- 5 |
| P3 | 当出现新的或加重的疼痛时，我会及时联系医护人员。 | 1 -- 2 -- 3 -- 4 -- 5 |
| P4 | 我会记录自己的疼痛情况（如使用疼痛日记）。 | 1 -- 2 -- 3 -- 4 -- 5 |
| P5 | 当出现止痛药的副作用时，我会向医生或护士寻求帮助。 | 1 -- 2 -- 3 -- 4 -- 5 |
| P6 | 疼痛时，我会尝试使用一些非药物方法（如听音乐、按摩）。 | 1 -- 2 -- 3 -- 4 -- 5 |
| P7 | 我会与家人讨论我的疼痛感受和治疗情况。 | 1 -- 2 -- 3 -- 4 -- 5 |
| P8 | 当我觉得疼痛减轻时，我会自己减少止痛药的用量或次数。(R) | 1 -- 2 -- 3 -- 4 -- 5 |
| 计分方式  （供研究者参考）正向条目1-5分计，反向条目(R)反向计分(5-1分)。总分越高，疼痛管理行为越规范。 | | |

**Cancer Pain Knowledge, Attitude, and Practice (KAP) Questionnaire for Patients in Southwest China**

Dear Patient Participant,

We are conducting a study on cancer pain management to better understand patients’ knowledge, attitudes, and practices regarding cancer pain. This questionnaire is anonymous. All information will be kept strictly confidential and used only for scientific research. Your honest responses are very important to us. The questionnaire takes approximately 10–15 minutes to complete.

Thank you very much for your support and participation.

**Part 1: Your Basic Information (Please check the appropriate box "√" or fill in the blank)**

Age: ______ years old

Gender: □ Male □ Female

Ethnicity: □ Han □ Tibetan □ Yi □ Miao □ Other ______

Education Level: □ Primary school or below □ Junior high school □ High school/Vocational school □ College/University or above

Place of Residence: □ Urban □ Town □ Rural

Cancer Diagnosis: □ Lung cancer □ Liver cancer □ Gastrointestinal cancer (esophagus, stomach, colorectal) □ Breast cancer □ Gynecological cancer □ Other _________

Cancer Stage: □ Early stage (I-II) □ Late/Advanced stage (III-IV)/Metastatic □ Unclear/Don't know

How severe is your pain right now? (0 means no pain, and 10 means the worst pain you can imagine)
0 -- 1 -- 2 -- 3 -- 4 -- 5 -- 6 -- 7 -- 8 -- 9 -- 10 (Please circle the number that best fits)

How long have you had this cancer pain?
□ Less than 1 month □ 1-3 months □ 3-6 months □ More than 6 months

Are you currently taking pain medication on a regular basis?
□ Yes □ No

**Part 2: Cancer Pain Knowledge (Please choose the answer you think is most appropriate)**

| No. | Question | Your Choice |
| --- | --- | --- |
| K1 | Cancer pain can be effectively controlled. | □ Yes □ No □ Don't know |
| K2 | Even when the pain is generally stable, sudden, brief, and severe pain (breakthrough pain) can occur. | □ Yes □ No □ Don't know |
| K3 | Taking pain medication on a regular schedule is more effective than waiting until the pain becomes severe. | □ Yes □ No □ Don't know |
| K4 | My doctor will adjust the dosage of my pain medication gradually to find the most suitable amount for me. | □ Yes □ No □ Don't know |
| K5 | Strong pain medications, like morphine, are unlikely to cause addiction when used under a doctor's guidance. | □ Yes □ No □ Don't know |
| K6 | The side effects of pain medication (e.g., constipation, nausea) can be prevented and managed. | □ Yes □ No □ Don't know |
| K7 | Besides medication, methods like listening to music, massage, and relaxation can also help relieve pain. | □ Yes □ No □ Don't know |
| K8 | Using a pain scale is an important way to help my doctor understand how much pain I am in. | □ Yes □ No □ Don't know |
| Scoring  (For researcher's reference) Correct answer = 1 point; Incorrect or "Don't know" = 0 points. A higher total score indicates better knowledge. | | |

**Part 3: Cancer Pain Attitude (Please circle the number that best reflects your true feelings)**

**Rating Scale: 1=Strongly Disagree, 2=Disagree, 3=Neutral, 4=Agree, 5=Strongly Agree**

| No. | Statement | Your Opinion |
| --- | --- | --- |
| A1 | I believe my pain can be well-relieved with treatment. | 1 -- 2 -- 3 -- 4 -- 5 |
| A2 | Enduring pain is a sign of strong willpower. (R) | 1 -- 2 -- 3 -- 4 -- 5 |
| A3 | Telling my doctor or family about my pain frequently will bother them. (R) | 1 -- 2 -- 3 -- 4 -- 5 |
| A4 | I am very worried that taking strong pain medication like morphine will lead to addiction, like drug abuse. (R) | 1 -- 2 -- 3 -- 4 -- 5 |
| A5 | I think it is important for my treatment to tell my doctor how much pain I have. | 1 -- 2 -- 3 -- 4 -- 5 |
| A6 | I am concerned that the side effects of pain medication will be worse than the pain itself. (R) | 1 -- 2 -- 3 -- 4 -- 5 |
| A7 | As long as it relieves the pain, I am willing to accept some side effects. | 1 -- 2 -- 3 -- 4 -- 5 |
| A8 | I will actively cooperate and use the pain medication prescribed by my doctor. | 1 -- 2 -- 3 -- 4 -- 5 |
| Scoring  (For researcher's reference) Positive items are scored 1-5. Reverse-scored items (R) are scored 5-1. A higher total score indicates a more positive attitude. | | |

**Part 4: Cancer Pain Practice (Behavior) (Please answer based on your actual situation in the past week)**

**Rating Scale: 1=Never, 2=Rarely, 3=Sometimes, 4=Often, 5=Always**

| No. | Practice | Your Frequency |
| --- | --- | --- |
| P1 | I proactively tell my doctor or nurse about the level and changes in my pain. | 1 -- 2 -- 3 -- 4 -- 5 |
| P2 | I strictly follow the schedule and dosage prescribed by my doctor for my pain medication. | 1 -- 2 -- 3 -- 4 -- 5 |
| P3 | When new or worsening pain occurs, I contact my medical team promptly. | 1 -- 2 -- 3 -- 4 -- 5 |
| P4 | I keep a record of my pain (e.g., using a pain diary). | 1 -- 2 -- 3 -- 4 -- 5 |
| P5 | When I experience side effects from pain medication, I seek help from my doctor or nurse. | 1 -- 2 -- 3 -- 4 -- 5 |
| P6 | When in pain, I try to use non-drug methods (e.g., listening to music, massage). | 1 -- 2 -- 3 -- 4 -- 5 |
| P7 | I discuss my feelings about pain and my treatment with my family. | 1 -- 2 -- 3 -- 4 -- 5 |
| P8 | When I feel my pain has lessened, I reduce the dose or frequency of my pain medication on my own. (R) | 1 -- 2 -- 3 -- 4 -- 5 |
| Scoring  (For researcher's reference) Positive items are scored 1-5. Reverse-scored items (R) are scored 5-1. A higher total score indicates more appropriate pain management behavior. | | |

This is the end of the questionnaire. Thank you again for your valuable time and support!
